# Cytocompatibility of Siloxane-Containing Vaterite/Poly(l-lactic acid) Composite Coatings on Metallic Magnesium

**DOI:** 10.3390/ma6125857

**Published:** 2013-12-12

**Authors:** Shinya Yamada, Hirotaka Maeda, Akiko Obata, Ulrich Lohbauer, Akiko Yamamoto, Toshihiro Kasuga

**Affiliations:** 1 Department of Frontier Materials, Nagoya Institute of Technology, Gokiso-cho, Showa-ku, Nagoya 466-8555, Japan; E-Mails: s.yamada.629@nitech.jp (S.Y.); obata.akiko@nitech.ac.jp (A.O.); 2 Center for Fostering Young and Innovative Researchers, Nagoya Institute of Technology, Gokiso-cho, Showa-ku, Nagoya 466-8555, Japan; 3 Dental Materials Lab, Dental Clinic 1, University of Erlangen-Nuremberg, Glueckstrasse 11, Erlangen 91054, Germany; E-Mail: lohbauer@dent.uni-erlangen.de; 4 Biometals Group, Biomaterials Unit, Nano-Bio Field, International Center for Materials Nanoarchitechtonics (MANA), National Institute for Materials Science (NIMS), 1-1, Namiki, Tsukuba 305-0044, Japan; E-Mail: yamamoto.akiko@nims.go.jp

**Keywords:** coating, magnesium, poly(l-lactic acid), vaterite (calcium carbonate), composite

## Abstract

Poly(l-lactic acid)-based films which include 60 wt % of vaterite (V) or siloxane-containing vaterite (SiV) were coated on a pure magnesium substrate, denoted by PLLA/V or PLLA/SiV, respectively, to suppress early corrosion and improve its cytocompatibility. Both coating films adhered to the Mg substrate with 2.3–2.8 MPa of tensile bonding strength. Soaking test for 7 days in α-modified minimum essential medium revealed that the morphological instability of the PLLA/V film caused a higher amount of Mg^2+^ ion to be released from the coating sample. On the other hand, in the case of the coating with the PLLA/SiV film, no morphological change even after the soaking test was observed, owing to the suppression of the degradation rate. In cell culture tests, the proliferation of mouse osteoblast-like cell (MC3T3-E1) was significantly enhanced by both coatings, in comparison with the uncoated magnesium substrate. The cell morphology revealed that a few less-spread cells were observed on the PLLA/V film, while more elongated cells were done on the PLLA/SiV film. The cells on the PLLA/SiV film exhibited an extremely higher alkaline phosphatase activity after 21 days of incubation than that on the PLLA/V one. The PLLA/SiV film suppressed the early corrosion and enhanced cytocompatibility on metallic magnesium.

## 1. Introduction

In recent years, metallic magnesium (Mg) and its alloys have become good candidates for metallic biomaterials because of their biodegradability [1,2] and mechanical properties [3]. They have high reactivity with water, and their degradation occurs in body fluid by corrosion. These properties are beneficial for biomaterials, such as vascular stents or orthopedic implants since they contribute to the avoidance of secondary surgery after healing and may lead to complete replacement of bone tissue [2]. Young’s modulus of Mg has been reported to be 41–45 GPa, which is similar to that of human cortical bone [4]. This value is lower than that of any other metallic biomaterials, including titanium alloys (e.g., Ti–6A–4V; 114 GPa) [3] or stainless steels (e.g., SUS 316L; 193 GPa) [3]. These mechanical properties might well decrease bone resorption around the implants.

However, there are some disadvantages of metallic Mg which must be overcome for clinical applications. Metallic Mg rapidly reacts with body fluid in the initial stage of implantation in body according to the Equation below:

(1) $$\mathrm{Mg}+2H_{2}O\to {\mathrm{Mg}}^{2+}+2{\mathrm{OH}}^{-}+H_{2}$$

According to this reaction, metallic Mg rapidly degrades, producing corrosion, hydroxyl ions, and bubbles of hydrogen gas around the surrounding tissues [5]. Extremely high local alkali concentration (pH > 9.0) on the surface is harmful for cells [6] and may inhibit cell adhesion or proliferation. Moreover, an accumulation of hydrogen gas around the implant was reported [5]. The bubbles of hydrogen gas were observed within a week and disappeared after 2–3 weeks. The bubbles formed vacant spaces around the Mg materials, and they inhibited the integration of implanted materials into body tissue.

Surface coatings containing biodegradable polymers were applied to the Mg substrate to improve corrosion resistance and cytocompatibility. Wong* et al.* [7] reported the coating of poly(ε-caprolactone) (PCL) onto a commercially available Mg alloy (AZ91). They concluded that the addition of a PCL coating on the implant reduced the corrosion rate. Meanwhile, Xu* et al.* reported spin-coating on the surface of the extruded, pure Mg with PCL or poly(l-lactic acid) (PLLA) [8]. They found that PCL and PLLA coatings provided significantly better cytocompatibility than uncoated Mg. Biodegradable polymers are expected to be one of the best candidates for coating on Mg substrates. However, due to the corrosion mechanisms of metallic Mg, such as evolution of hydrogen gas, partial delamination between coating film and Mg substrate might be induced, subsequently burst into degradation [9]. The partial delamination would affect its cytocompatibility. Therefore, the polymer coatings are required to possess enough bonding strength to avoid the delamination.

Currently, composite materials, which consist of the polymers with the inorganic particles of bioactive materials such as Bioglass^®^, calcium phosphate or hydroxyapatite, are being investigated for improving bone integration [10,11]. Asselin* et al.* suggested that the calcium ion released from Bioglass^®^ has a stimulatory effect on the activation of osteoblasts [12]. We have focused on vaterite, which is one of bioresorbable polymorph of calcium carbonates. Vaterite is the most soluble among the calcium carbonate polymorphs and releases calcium ions, which influence the activities, such as the proliferation, differentiation, and so on, of osteoblastic cells [13]. Our group has been developing PLLA-based composites containing vaterite (V) particles [14,15]. This material is denoted “PLLA/V” hereafter. In our previous work, a PLLA/V film formed a bone-like apatite layer on its surface after soaking in simulated body fluid [14], and the number of human osteoblasts on the PLLA/V film after 1 week of the incubation was greater than that on the PLLA film [16].

We also have been developing siloxane-containing vaterite/PLLA hybrid materials [17,18], which are hereafter denoted “PLLA/SiV”. Siloxane-containing vaterite (SiV) having the ability to release silicate and calcium ions was prepared by the addition of aminopropyltriethoxysilane as a silicate source during the carbonation process. In our earlier work [19], it was demonstrated that the cell number and alkaline phosphatase (ALP) activity of mouse osteoblast-like cells (MC3T3-E1 cells) cultured on PLLA/SiV significantly increased in comparison with cells cultured on a control sample without the releasability of silicate ions. Xynos* et al.* also reported that the stimulatory effect on the enhancement of bone formation by the silicate and calcium ions released from Bioglass^®^ [20]. PLLA/V- or PLLA/SiV-coatings is expected to lead to the improvement of cytocompatibility.

To test this hypothesis, PLLA/V and PLLA/SiV were coated on a pure Mg surface, and then their corrosion resistances and cytocompatibilities were evaluated. Local bonding strength of the coating films was also measured.

## 2. Results and Discussion

### 2.1. Morphologies of the Coating Films

**Figure 1:**
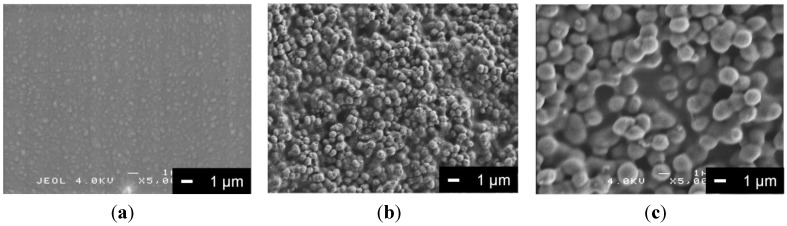
SEM images of (**a**) poly(l-lactic acid (PLLA)-coating; (**b**) PLLA/vaterite (V)-coating; and (**c**) PLLA/siloxane-containing vaterite (SiV)-coating.

Figure 1 shows scanning electron microscope (SEM) images of the samples. No abrasive tracks could be observed on any of the Mg substrate surfaces. This implies that the Mg surface was successfully coated with PLLA, PLLA/V or PLLA/SiV. V or SiV particles in the PLLA/V- and PLLA/SiV-coating were covered with a PLLA layer, but the layer may have been very thin since the shapes of the particles were distinct. The thickness of each coating film, estimated by measurement from the cross-sectional SEM images, and the average surface roughness (*Ra*) values of each coating surface are shown in Table 1.

**Table 1:** Thickness and surface roughness of the coatings on Mg.

Sample Code	Thickness (µm)	Roughness, *Ra* (µm)
PLLA	1.8 ± 0.2	0.08 ± 0.01
PLLA/V	3.0 ± 0.1	0.19 ± 0.01
PLLA/SiV	5.3 ± 0.4	0.40 ± 0.00

The PLLA-coating was found to be the thinnest film at 1.8 µm, while the PLLA/SiV-coating was the thickest one at 5.3 µm. The thickness of a film applied by the spin-coating method depends on the concentration and viscosity of polymer solutions [21]. The *Ra* value of the PLLA/V-coating was 0.19 µm, while that of the PLLA/SiV-coating was 0.40 µm. The particle size of V or SiV blended into the PLLA solution may influence the viscosity of the solution and the surface roughness of the coating.

### 2.2. Tensile Bonding Strength

**Figure 2:**
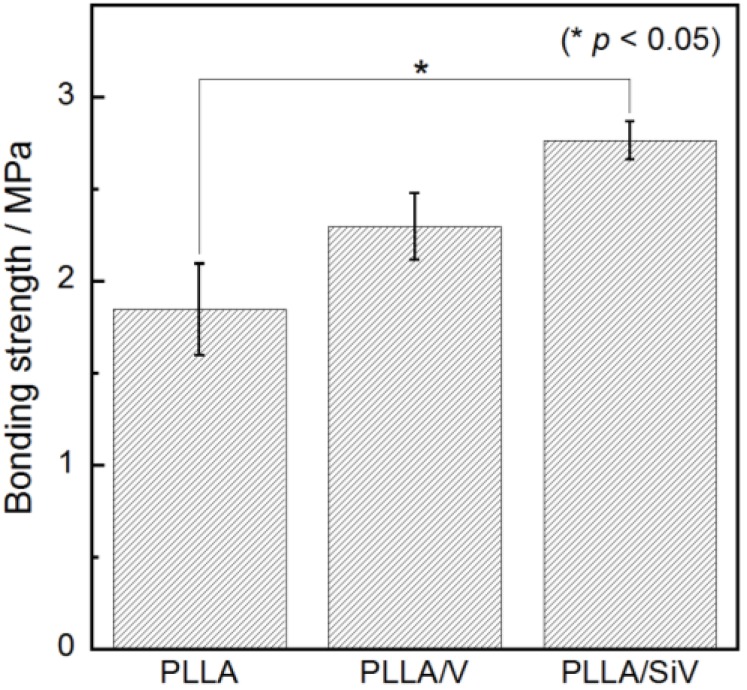
Tensile bonding strength between the coatings and Mg substrate. (mean ± standard error of the mean(S.E.M.); * *p* < 0.05 as compared to PLLA coating by *t*-test).

The bonding strengths between the coatings and Mg substrate are exhibited in Figure 2. This is our original method to evaluate the tensile bonding strength of the coatings. The fracture was found to certainly occur between the coatings and the substrate, since the surface of metallic Mg had been exposed after the test. PLLA/SiV-coating indicated the highest bonding strength (2.8 MPa in average) among the samples. The strength of PLLA/V-coating was 2.3 MPa, which was slightly higher than that of PLLA-coating (1.8 MPa), without significant difference. The strengths tended to increase by the additives in the coating. Xu *et al.* reported the bonding mechanism was influenced by the molecular weight of the polymer coatings [8]. Low molecular-weight polymers have more free ends of the polymer chains compared with high molecular-weight ones, which provide a large number of free carboxyl groups for electrostatic intermolecular interaction between polymer chain and the Mg substrate surface. The molecular weights of PLLA/V and PLLA/SiV were 46 kDa and 82 kDa, respectively, which were lower than that of PLLA (90 kDa).

According to this result, PLLA/V might show high bonding strength. However, the bonding strength of PLLA/SiV was the highest, while its molecular weight was almost same as that of PLLA. The differences in film thickness and fracture behavior between the coating films may also concern to the bonding strengths measured by this method. The further discussion is now in progress.

### 2.3. Degradation of Mg Substrates and the Coating Films

**Figure 3:**
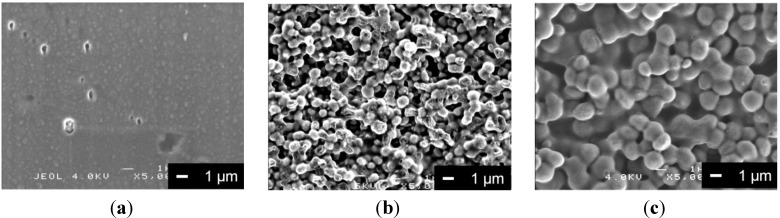
SEM images of (**a**) PLLA-coating; (**b**) PLLA/V-coating; and (**c**) PLLA/SiV-coating after soaking in α-MEM at 37 °C for 7 days.

Figure 3 shows the surface morphologies of the coatings after soaking in α-MEM for 7 days. No peeling of the coating films was observed during the soaking in the culture medium for all of the samples. As shown in Figure 3a, some pores of 0.5 μm in diameter were observed on the PLLA-coating surface. These pores may have originated from the hydrolysis of PLLA. On the other hand, many pores with the size of 1–2 μm in diameter were observed on the PLLA/V-coating surface (Figure 3b). The surface morphology changed drastically after soaking, compared with samples before soaking. These pores might have formed due to the degradation of the PLLA matrix in PLLA/V and the detachment of V particles from the coating. In the case of the PLLA/SiV-coating, no significant change was observed during the soaking (Figure 3c). The SiV surface in the PLLA/SiV-coating might be covered with a larger amount of PLLA phase, which led to a suppression of the degradability of the coating. Another possible reason is the difference in dissolution behavior for the inorganic particles, since V has a smaller particle size than SiV.

**Figure 4:**
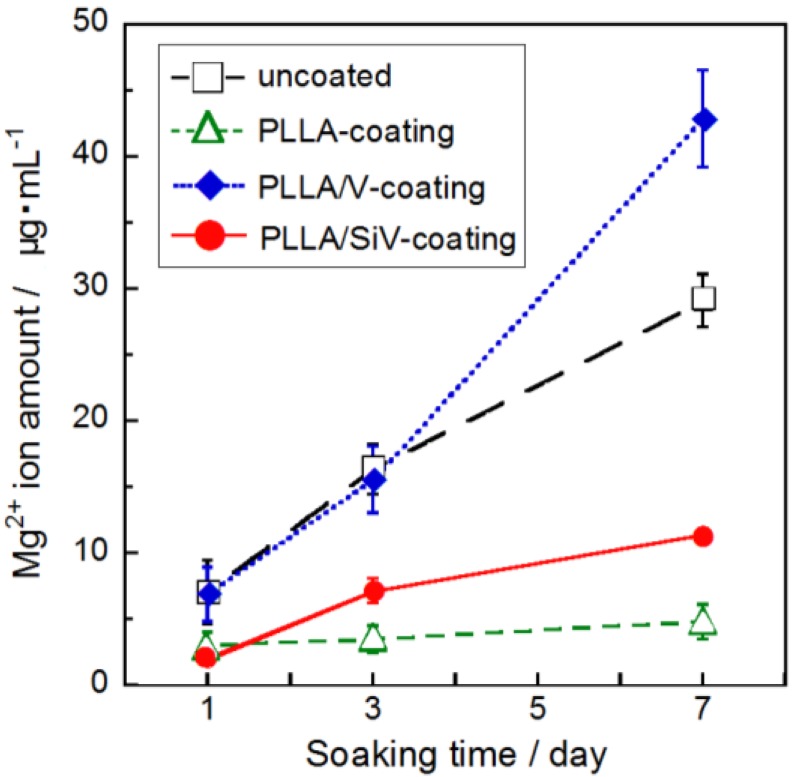
Mg^2+^ ion concentrations dissolved from (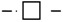) uncoated, (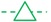) PLLA-coating, (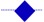) PLLA/V-coating and (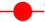) PLLA/SiV-coating. (*n* = 3).

The amounts of Mg^2+^ ion released from the samples into the α-MEM are shown in Figure 4. The media were not replaced during the soaking. Up to 30 μg/mL of Mg^2+^ ions were released from the uncoated sample (pure-Mg substrate) over 7 days. The corrosion of the Mg substrate continued during this period, since the amount of the released Mg^2+^ ion continued to increase. In comparison with the uncoated sample, the PLLA-coating showed lower amounts of Mg^2+^ ion released from the samples over 7 days. Even after soaking for 7 days, the amount of Mg^2+^ ion released was about 5 μg/mL. Although, some pores were found in the PLLA-coating as shown in Figure 3a, they induced almost no severe corrosion of Mg. Moreover, the PLLA/SiV-coating suppressed the amount of Mg^2+^ ion released up to 11 μg/mL over 7 days. These coatings suppressed the corrosion of metallic Mg under these experimental conditions. However, the PLLA/V-coating released almost the same amount of Mg^2+^ ion as the uncoated Mg substrate until day 3. After soaking for 7 days, it released higher amount of Mg^2+^ ion compared with the uncoated Mg substrate. The formation of larger pores on the PLLA/V-coating surface (Figure 3b) seems to cause an increase in the amount of Mg^2+^ ion released from the sample. The local environment, such as the amount of surrounding ions, influences the corrosion of metallic Mg [22]. Ca^2+^ ions released from the PLLA/V-coating owing to the degradation of vaterite may accelerate the corrosion of the Mg substrate.

### 2.4. Cell Proliferation and Morphology

**Figure 5:**
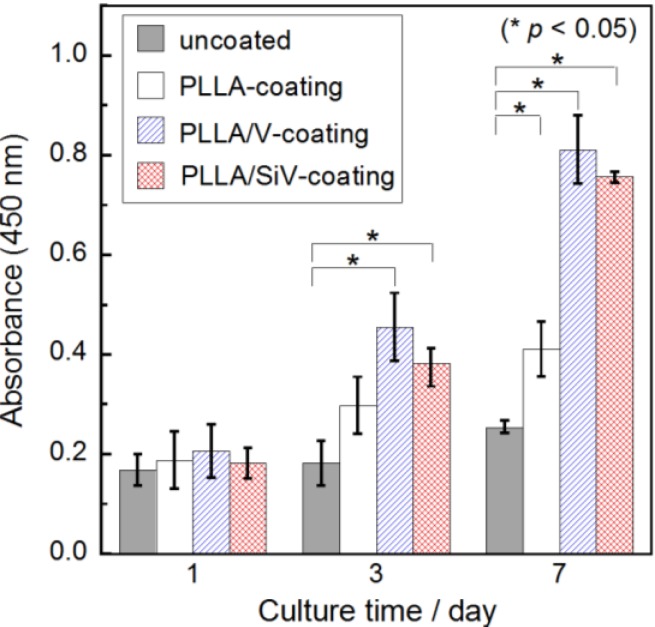
Absorbance as a function of cell numbers for MC3T3-E1 cells cultured on uncoated Mg, PLLA-coating, PLLA/V-coating and PLLA/SiV-coating. (*n* = 3) (mean ± S.E.M.; * *p* < 0.05 as compared to uncoated by *t*-test)

Figure 5 shows the proliferation of MC3T3-E1 cells cultured on each of the samples. The absorbance corresponds to the number of living cell on the sample. The initial concentration of cells seeded on the samples (6000 cells/mL) corresponds to an absorbance of approximately 0.2. Under these experimental conditions, the cell numbers kept increasing during 7 days of incubation for all samples. There were no significant differences in living cell numbers among the samples after 1 day of incubation. The cells may have comparable abilities for adhering to each coating surface. After 3 days, the number of living cells on the uncoated sample was almost the same as the number of cells cultured after 1 day. The cells continued to slightly proliferate for 7 days, and there was no significant change in cell number. The number of living cells on the PLLA-coating was larger than that on the uncoated Mg substrate after 7 days of incubation. This implies that the biodegradable polymers are capable of promoting cell proliferation, which is consistent with a previous report [8]. The number of cells on the PLLA/V- and PLLA/SiV-coatings were significantly higher than that on the uncoated samples after 3 and 7 days of incubation. In general, surface roughness is an important factor for cellular activities such as adhesion, proliferation and gene expression. The surface morphology and roughness on the PLLA/SiV-coating are considered to relate to this cell proliferation ability (Figure 1b and Figure 3b and Table 1). On the other hand, although the PLLA/V-coating has smoother surface, there was no significant difference between the PLLA/V- and PLLA/SiV-coatings. It has been also reported that the crystallinity of polymer relates to cellular proliferation [23,24]. The crystalline PLLA substrate exhibited lower growth rate of cells than amorphous substrate. However, the crystallinity of PLLA, PLLA/ V and PLLA/SiV were from differential scanning calorimetry (DSC) analysis to be 14%, 9% and 12%, respectively. The cells on the samples proliferated independently of the crystallinity of the coatings. The ions released from the PLLA/V-coating may stimulate cell proliferation on the coating. There may be a large amount of Ca^2+^ ions, which are known to enhance cell proliferation on the PLLA/V-coating, because the coating seems to degrade as shown in Figure 3b. The effects of Mg^2+^ ion on cellular activities are still not fully understood. There are some hypotheses regarding the reaction of osteogenic cells. For example, Diba* et al.* reported the stimulatory effects on the growth and development of osteoblasts [25]. Ca^2+^ and Mg^2+^ ions released from the PLLA/V-coating (Figure 4) might enhance cell proliferation on its surface.

**Figure 6:**
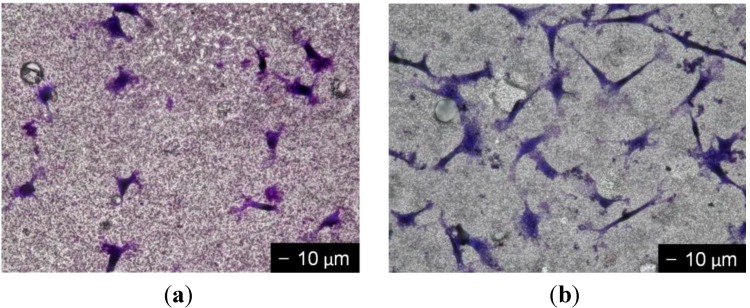
Optical micrographs documenting the cell morphologies of MC3T3-E1 cells cultured for three days on (**a**) PLLA/V-coating and (**b**) PLLA/SiV-coating.

The optical micrographs of MC3T3-E1 cells cultured for three days and stained with Giemsa’s solution are shown in Figure 6. MC3T3-E1 cells begin to adhere to the surface within 1 day of seeding, and then begin to proliferate. In this work, the cell morphologies during the proliferation stage were evaluated. The cell morphology of the PLLA/V-coating was different from that of PLLA/SiV-coating. The spindle-like shape is the preferred morphology of MC3T3-E1 cells. Only a few less-spread cells or circular cells were observed on the PLLA/V-coating. In comparison with this, more elongated cells were seen on the PLLA/SiV-coating. The cell morphologies were quantified using the aspect ratio. The cell aspect ratios were estimated from optical micrographs using a software (Image J; NIH, US National Institutes of Health, Bethesda, Maryland, MD, USA) as the ratio of the major axis to the minor one. It was found that the cells on the PLLA/V-coating had an average aspect ratio of 1.4 ± 0.3, while those on the PLLA/SiV-coating had an average aspect ratio of 3.2 ± 0.7. The adhesion and spreading of osteoblasts are related to the surface characteristics such as roughness, wettability or functional groups [26,27]. We believe that the surface roughness (Table 1) is one of the reasons for the difference in cell morphologies between the PLLA/V- and PLLA/SiV-coatings. The surface instability of the PLLA/V-coating (Figure 3b) might also inhibit cell spreadings, even though their living cell numbers were comparable.

### 2.5. ALP Activity of the Cells

**Figure 7:**
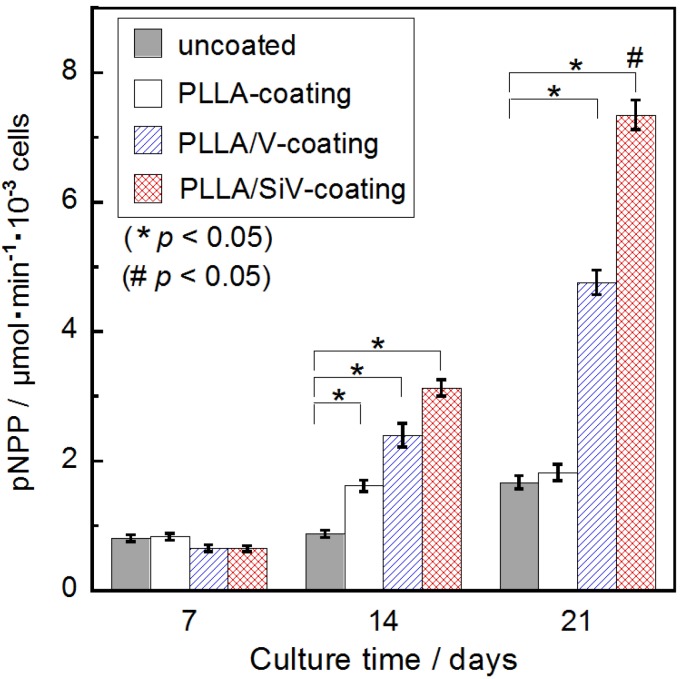
Alkaline phosphatase (ALP) activities of MC3T3-E1cells cultured on the samples. (*n* = 3) (mean ± S.E.M.; * *p* < 0.05 as compared to uncoated by *t*-test, # *p* < 0.05 as compared to other three samples by Tukey’s multiple comparison test).

Figure 7 indicates the ALP activities of MC3T3-E1 cells cultured on the coatings for 7, 14 and 21 days. After incubation for 14 days, the ALP activities of the MC3T3-E1 cells cultured on the coating samples were higher than those of the MC3T3-E1 cells cultured on the uncoated samples. Moreover, after 21 days of culture, the cells on the PLLA/SiV-coating displayed a much higher ALP activity than any of the other samples. This suggests that the differentiation of MC3T3-E1 cells was strongly enhanced on the PLLA/SiV-coating. PLLA/SiV film successfully prevented rapid degradation of the Mg substrate (Figure 4). Eventually, protection from rapid degradation might enhance cellular differentiation. Cell morphologies are related to gene expression [28]. The morphologies of the cells shown in Figure 6 seem to relate to cell activities such as cell differentiation (Figure 7). Moreover, silicate ions are known to promote osteogenic cell differentiation [29]. We expect that silicate ions begin to be released from the PLLA/SiV-coating and affect cell differentiation after 7 days.

## 3. Experimental Section

### 3.1. Sample Preparation

A commercially available pure Mg rod (diameter: 9.5 mm, purity: 99.95%, Nilaco, Tokyo, Japan) was cut into disks with a thickness of 2.5 mm using a low-speed saw with a diamond blade, and then polished with a 1200-grit SiC abrasive paper (15 μm, Noritake Coated Abrasive, Aichi, Japan) for use as substrates. The discs were ultrasonically washed twice in acetone for 5 min. All of the Mg samples were covered with resin except on their top side.

PLLA/V and PLLA/SiV, containing 60 wt % (*i.e.*, 47 vol %) V or SiV, respectively, were prepared by a melt-blending method at 180 °C for 10 min using PLLA (LACEA^®^, molecular weight: 120 kDa, Mitsui Chemicals, Tokyo, Japan) and V (0.5 μm-diameter, Yabashi Industries, Gifu, Japan) or SiV (Si content; 2.8 wt %, 1.5 μm-diameter, Yabashi Industries, Gifu, Japan) as starting materials. The molecular weights of PLLA in the PLLA/V or PLLA/SiV were measured by gel-permeation chromatography (GPC; LC-20, Shimadzu, Kyoto, Japan). Differential scanning calorimetry (DSC) measurements were performed using ThermoPlus (DSC-8230; Rigaku, Tokyo, Japan) at a heating rate of 5 °C·min^−1^. The crystallinity of the PLLA matrix was calculated according to the report of Iafisco* et al.* [24].

These materials were dissolved in chloroform to obtain solutions for spin-coating. The PLLA concentration in each solution was adjusted to 4 wt %. Each solution (100 μL) was added dropwise for 90 s on the top-side surface of a Mg substrate mounted on a spin-coater spinning at 5000 rpm. Hereafter, the samples, which were coated with PLLA/V or PLLA/SiV, are denoted by “PLLA/V-coating” or “PLLA/SiV-coating”. An uncoated, polished Mg substrate was used as a control, which is denoted by ‘uncoated’. A sample substrate was also coated with PLLA without inorganic particles, kneaded by the melt-blending method under the same condition, which is denoted by “PLLA-coating”.

Surface and cross-section of samples were observed with a scanning electron microscope (FE-SEM; JSM-6301F, JEOL, Tokyo, Japan). The average surface roughness (*Ra*) was examined using a surface roughness tester (SURFCOM 1400-D, Tokyo Seimitsu, Japan). At least 5 samples were tested for each type of material.

### 3.2. Tensile Bonding Strength Test

**Figure 8:**
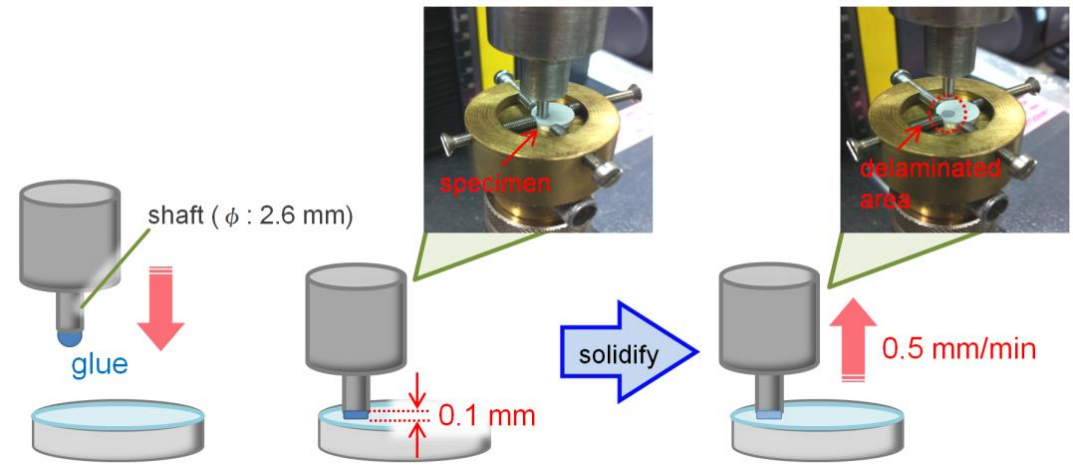
Schematic drawing of a measuring method for tensile bonding strength between the coating and Mg substrate.

Figure 8 illustrates schematic drawing of a testing method for measuring bonding strength between the coating and Mg substrate. The specimen was mounted on a brass jig, and subsequently an aluminum shaft (diameter: 2.6 mm) was bonded on the surface of the coating with cyanoacrylate glue. The distance between the coating surface and shaft was spaced to be 0.1 mm. After 5 min for the solidification of the glue, the aluminum shaft was slowly moved up and the bonding strength was measured with a tensile strength machine (X force HP 1kN, Zwick Roell, Ulm, Germany) with a tensile rate of 0.5 mm/min. At least 20 points for each kind of the coating were tested.

### 3.3. Soaking Test in a Culture Medium

All samples were sterilized with ethylene oxide gas (EOG) at 44 °C for 24 h. Each sample was then soaked in 27.5 mL of α-modified minimum essential medium (α-MEM; MEMα with l-glutamine and phenol red, Wako, Japan) containing 10% fetal bovine serum (FBS) and incubated in 5% (v/v) CO_2_ at 37 °C for 1–7 days. To estimate the corrosion rate of the samples, the amount of Mg^2+^ ions released from the samples was measured by inductively coupled plasma atomic emission spectroscopy (ICP-AES; ICPS-500, Shimadzu, Kyoto, Japan). This measurement was repeated for a total of three repetitions. The sample surfaces were observed with FE-SEM after soaking in α-MEM for 7 days.

### 3.4. Cell Culture Test

Mouse-calvaria-derived osteoblast-like cells (MC3T3-E1 cells) were seeded on the samples placed in a glass container (*ϕ*: 60 mm, height: 45 mm), after sterilization with EOG, in 27.5 mL of α-MEM at a cell density of 6000 cells/mL. The samples were placed in a CO_2_ incubator for 1–7 days. The proliferation rate of MC3T3-E1 cells was evaluated after treatment using a Cell Counting Kit-8 (CCK-8; Dojindo, Kumamoto, Japan), following its instructions. The numbers of live cells on the samples were colorimetrically estimated by measuring the absorbance of the resulting media at 450 nm with a microplate scanning spectrophotometer (Sunrise Remote; Tecan, Kanagawa, Japan). The ALP activity of the cells cultured on the samples was analyzed using a *p*-nitrophenyl phosphate (pNPP) tablet sets (Lab Assay ALP Kit; Wako, Japan). ALP activity was evaluated after measuring the absorbance of *p*-nitrophenol product formed at 405 nm with the spectrophotometer. The value of APL activity was expressed as μmol of pNPP/min/cells. The samples were fixed with a 2.7% (v/v) glutaraldehyde solution for 10 min, and then stained with Giemsa’s solution. The stained samples were observed with an optical microscope (Biorevo BZ-9000; Keyence, Osaka, Japan). Three parallel samples of each coating were cultured.

### 3.5. Statistical Analysis

Data were presented as the mean ± standard error of the mean (S.E.M.). Statistical analysis was performed using Student’s *t*-test and single-factor ANOVA (SPSS 21 software; IBM, Armonk, NY, USA) followed by Tukey’s multiple comparison test. Values of *p* < 0.05 were considered to be significant.

## 4. Conclusions

A pure Mg substrate was coated with a PLLA film or with two types of PLLA-based films,* i.e.*, PLLA/V and PLLA/SiV, which contain different inorganic particles; (V and SiV) by a spin-coating method to evaluate the effects of the coatings on corrosion protection and the cytocompatibility of the materials. Addition of inorganic particles in the coatings improved bonding strength with Mg substrate. The surface observation with SEM demonstrated the morphological stability of the PLLA- and PLLA/SiV-coatings after soaking in α-MEM for 7 days. These coatings controlled the release of Mg^2+^ ion from the samples. In contrast, many pores with sizes of 1–2 μm in diameter formed on the PLLA/V-coating during the soaking, and this coating induced severe degradation of Mg. Cell culture tests indicated that MC3T3-E1 cells proliferated well on the PLLA/V- and PLLA/SiV-coatings during 7 days of incubation. Only a few less-spread cells or circular cells were observed on the PLLA/V-coating, while more elongated cells were found on the PLLA/SiV-coating. The cells attached on the PLLA/SiV-coating exhibited the highest ALP activity after 21 days of incubation. The PLLA/SiV-coating inhibited the initial rapid corrosion of metallic Mg, and enhanced the proliferation and differentiation of the cells.
